# Optimization of thermal properties of palm fiber and nanofillers reinforced epoxy nanocomposite

**DOI:** 10.1038/s41598-025-03474-x

**Published:** 2025-07-01

**Authors:** Suresh Thirupathi, Venkatachalam Gopalan, Elango Mallichetty

**Affiliations:** 1 School of Mechanical Engineering, Vellore Institute of Technology, Chennai, Tamilnadu 600127 India; 2 Centre for Advanced Materials and Innovative Technologies, Vellore Institute of Technology, Chennai, Tamilnadu 600127 India

**Keywords:** Palm fruit fiber, Sodium hydroxide treatment, Thermal conductivity, Nanofillers, RSM-BBD, Materials science, Biomaterials, Nanoscale materials

## Abstract

In recent times, the growing economic concerns and the rising cost of synthetic fibers have shifted focus to natural fiber reinforced composites, which are evident in the current literature. It is important to understand the thermal properties of these materials for functional applications. This study investigates the thermal conductivity of epoxy composites reinforced with sodium hydroxide (NaOH)-treated palm fruit fiber, a rarely used natural resource, with the addition of nanofillers, multi-walled carbon nanotubes, hexagonal boron nitride (h-BN), and aluminium oxide (Al_2_O_3_) used at 1 wt.%. The hand layup method is used to fabricate nanocomposites with varying palm fiber content (1–5 wt.%) with mesh size (75–225 µm) and nanofiller. The prepared nanocomposite samples are measured for thermal conductivity. SEM analysis manifests that alkali treatment increases the surface morphology of fiber as cleansed from impurities, which in turn increases bonding with the epoxy matrix at the interface. RSM was used to optimize these parameters, specifically the Box-Behnken Design (BBD) of RSM, with ANOVA analysis using Design-Expert 13 software to generate a strong regression model. The results demonstrate a maximum thermal conductivity of 1.54 W/m·K with 1 wt.% of palm fiber, 75 µm mesh size and 1% h-BN. This research emphasizes the promise of epoxy composites based on palm fibers for applications in thermal management in areas such as electronic devices, and circuit boards while contributing to the development of novel and efficient material solutions.

## Introduction

Evaluating the thermal properties of composite materials is crucial relevance because they find extensive use in varied applications necessitating good heat management, efficient thermal insulation and optimised heat transfer capacities^1–4^. The thermal conductivity of composite materials can vary greatly depending on various parameters, including the intrinsic qualities of the constituent materials, fibre orientation, composite construction method and filler incorporation^5^. Natural fibers have gained significant attention as bio-reinforcements in polymer composites due to their ability to enhance mechanical strength^6,7^, thermal stability^8,9^ and wear resistance^10,11^. Palm fibre epoxy composites have gained much interest in recent years due to their potential in lightweight applications.

Palmyra palm fibre reinforced epoxy composites possess superior thermal conductivity, making them appropriate for various applications. Research indicates that adding palmyra fibres to epoxy resin improves mechanical, physical, tribological, thermal and acoustic qualities^12^. Palmyra palm fibre composites possess diverse heat conductivities depending on fibre composition and fabrication procedures. Research on palm fibre reinforced polyester composites (PFRP) revealed that thermal conductivity of the composite rises through larger fibre percentages, the actual results fitting closely with theoretical predictions^13^. Polymer composites, reinforced with natural fibres like Date Palm Fiber (DPF), demonstrated the potential to improve thermal conductivity. Researchers investigated different methods to boost thermal characteristics. Composites made of DPF and poly (β-hydroxybutyrate) showed that as the amount of DPF increases, so does the thermal conductivity, achieving a data of 0.112 W/(m.K)^14^.

After adding different fillers like boron nitride (BN), magnesium oxide (MgO), multiwalled carbon nanotubes (MWCNTs) and AlN whiskers, epoxy composites showed improved thermal conductivity. Researchers indicated that thermal conductivity of cellulose nanofiber (CNF) aerogels can be raised by 389.5% by including fillers such as MWCNTs^15^. On the other hand, epoxy composites with hybrid fillers containing BN and MgO can achieve 1.97 W/(m·K) of thermal conductivity^16^. Further, a thermal conductivity of 1.65 W/(mK) in epoxy composites can be achieved through the synergistic impact of physically blending fillers and building primary-secondary thermal conductivity networks utilising BN^17^.

Utilizing natural cotton as a precursor, Carbon Fiber Aerogel (CFA) and SiC@CFA composite materials exhibited excellent thermal conductive properties even with minimal filler addition. The thermal conductivity of SiC@CFA embedded within an Epoxy matrix was recorded at 0.404 W/(m·K) at a filler of 1.85 wt%^18^. Integrating 3D graphene aerogel into natural rubber composites yielded thermal conductivity of 0.891 W/(m·K) at a 25 wt.% graphene loading, indicating promising outcomes for thermal management uses^19^. The mixture of natural fibers and hexagonal boron nitride (hBN) boosted thermal conductivity dramatically, with a composite of hBN, kenaf fiber and epoxy reaching 6.418 W m^−1^ K^−1^^20^. Eucalyptus fiber-reinforced polyester composites showed a small decrease in heat stability, indicating potential practical application restrictions^21^. A composite of BN, lignosulfonate and natural rubber thermal conductivity (1.17 W m^−1^ K^−1^) had the potential for heat management in electronics applications^22^. The addition of palmyra fibres improved certain composites’ capacity to dissipate heat because of their increased thermal conductivity^23^. Additionally, fillers such as Al_2_O_3_ and BN were used to create a three-dimensional thermal network, which significantly increased thermal conductivity of epoxy composites. This made materials perfect for thermal management applications in the energy and electrical encapsulation fields^24,25^.

The inclusion of a composite nanofiller made from hexagonal boron nitride (hBN), carbon nanotubes (CNTs) and aluminium oxide (Al_2_O_3_) nanoparticles could significantly increase the thermal conductivity of palm fiber epoxy composites. Researchers indicated that integrating hBN and CNTs into the epoxy resin substantially enhances thermal conductivity. Specific filler concentrations were reported to achieve thermal conduction performance of 10.18 W/(m K)^26,27^. Including hBN and Al_2_O_3_ within epoxy composites was shown to elevate their thermal conductivity to 1.72 W·m^−1^·K^−1^, representing a significant enhancement over pure epoxy composites. This enhancement suggested that combining hBN, CNTs and Al_2_O_3_ nanofillers with palm fiber epoxy composites could significantly improve their thermal conductivity. Such composites, used in electronic devices^28,29^, could potentially be valuable for applications necessitating effective heat dissipation. The addition of hexagonal boron nitride (hBN), carbon nanotubes (CNTs) and aluminium oxide (Al_2_O_3_) boosted thermal conductivity with improved thermal stability, reduced coefficient of thermal expansion and modified electrical properties. These attributes made the composites ideal for use in electronic packaging. The synergistic effect of these nanofillers forms an effective thermal conduction network, resulting in high thermal conductivity and reduced electrical conductivity, positioning these materials as excellent options for heat management in electronic components.

The impact of the natural fibre content by weight and the mesh size of fiber on the heat transfer performance of composites was extensively documented in the literature. Studies showed that the transverse conductivity heat transfer of unidirectional composites increases with a higher content of bamboo fibres, whereas it decreases when abaca fibres are used. This variation underscored the importance of the internal lumen structure of the fibres in influencing their thermal characteristics^30,31^. The research highlighted a correlation between the effective transverse thermal conductivity and the geometrical ratio of the natural fiber-polymer composite. It pointed out the significant influence of the size of the fiber lumen on the composite’s thermal conductivity^32^.

Recent research revealed that some polymers possess surprisingly high levels of thermal conductivity, rivalling those of inferior metals or even silicon. This finding contradicts the conventional perception of polymers as primarily thermal insulators. Enhancing the polymers’ thermal conductivity and their nanocomposites emerged as a key area of research, driven by the challenges associated with polymers’ inherently low thermal conductivity in uses such as heat exchangers and electronic packaging^33^. The addition of hexagonal boron nitride nanoparticles into high-density polyethylene using plasma treatment demonstrated beneficial effects, considerably augmenting the heat conductivity of the nanocomposites in contrast to the pure polymer specimen^34^. Polymer nanocomposites, featuring inorganic nanoparticles evenly dispersed within the polymer matrix, offered a distinctive combination of inorganic and polymer properties. These materials were utilized in various applications across mechanics, thermal conductivity, electrical conductivity, optics and other areas^35^.

This research uses palm fruit fiber, an agricultural by-product, to enhance thermal conductivity of epoxy-based nanocomposites. The study investigates the sodium hydroxide (NaOH) treated palm fruit fiber enhanced thermal properties of the material. It integrates natural fiber reinforcement with advanced nanofillers like h-BN, MWCNTs and Al_2_O_3_ to boost thermal performance. This work aims to create materials that offer better thermal management for use in electronics, automotive applications and energy storage. By utilizing palm fruit fibers (PFF), which are often overlooked as waste, this research enhances material properties and contributes to waste reduction. The study seeks to develop high-performance composites that provide superior thermal conductivity for industrial heat management systems by optimising fibre content, mesh size and the types of nanofillers used.

## Materials and methods

### Material

**Fig. 1:**
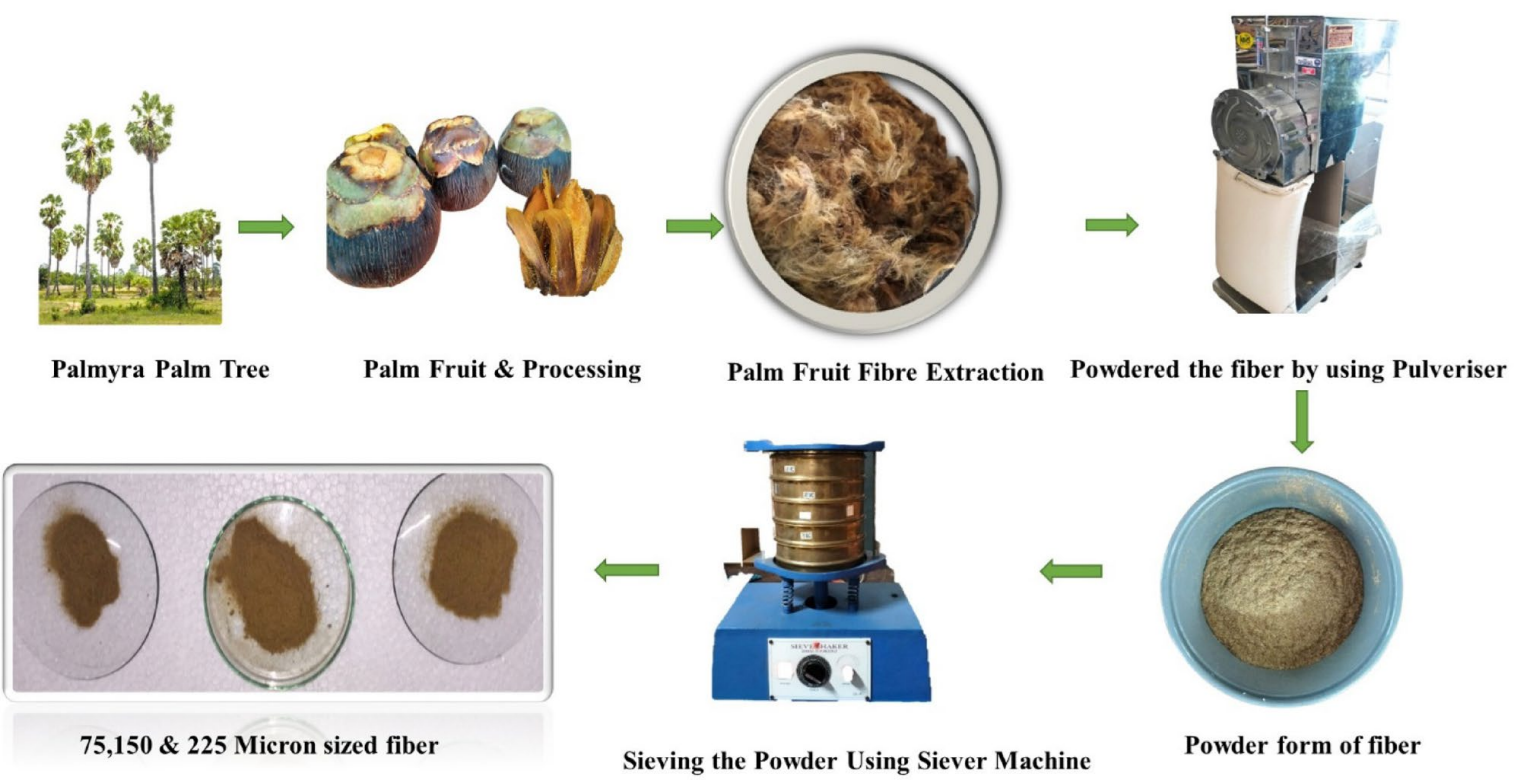
Palm fruit fiber processing.

Epoxy resin, sodium hydroxide (NaOH) and hardener (HY 951) are purchased from Hernba Instrument & Engineers, Chennai, Tamil Nadu, India. The Palmyra palm fruit fibres are collected from Tirunelveli, Tamilnadu. Palm fibres are chopped into 20-mm lengths, crushed and sorted using a sieve apparatus. Palm particles of mesh sizes from 75 to 225 microns are used as reinforcing elements in composites. Figure 1 depicts prepared palm fibres and particles. Nanofillers MWCNT & alumina are purchased from the Sisco Research Laboratories Pvt. Ltd, Mumbai, India and Nano h-BN is purchased from Nanoshel supplier. The nanofiller specifications are mentioned in Table 1.

**Table 1:** Specifications of nanofillers.

Filler	Size (nm)	Structure	Colour	Purity (%)	Density (g/cm^3^)
MWCNT	10–20	Tube	Black	95	2.1
h-BN	60	Hexagonal	White	99.9	2.25
Al_2_O_3_	20–30	Spherical	White	99.9	3.95

### NaOH-modified palm fiber

**Fig. 2:**
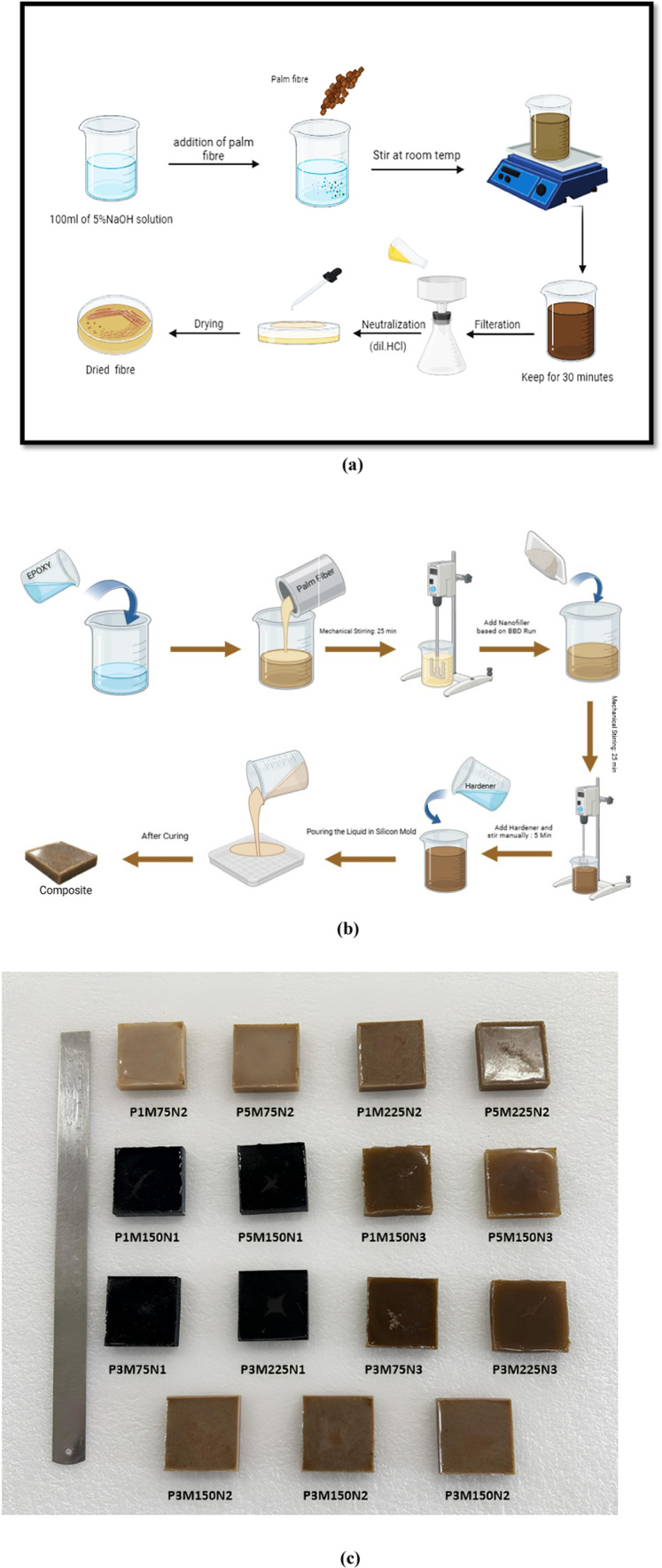
(**a**) Alkaline treatment of palm fiber. (**b**) Fabrication technique of composite material. (**c**) Fabricated samples of nanocomposite materials.

Alkali treatment of organic fibers is a well-established technique for modifying the structural formula of cellulose. Regular water is used for about 2 h to wash away the palm particles. After that, the palm particles are dried at room temperature for one day and then placed in drying oven at 90 °C for four hours. Then 5% sodium hydroxide (NaOH) solution is prepared by mixing it with distilled water and the palm particles are soaked in this solution for 30 min as illustrated in Fig. 2 (a). To further dry the PF particles, they are placed in an oven at 100 °C for 18 h^36–39^.

1 $${\text{Natural Fiber} \, -\text{ O}}^{-}{H}^{+ }+{Na}^{+ }-{OH}^{- }\to Palm Fiber-{O}^{-} {Na}^{+}+ {H}_{2}O$$

### Fabrication of nano-composites

The palm fibre-reinforced epoxy composites are fabricated using a hand layup procedure and a silicon mould size of 50 mm × 50 mm × 10 mm as shown in Fig. 2b. Nanocomposites are formed by mixture of used polymer and hardener in a 10:1 weight ratio. Silicone spray is applied on mould surfaces to remove composite plates after hardening. This research produces nanocomposites by blending dried palm particles and various fillers (such as nano MWCNT, h-BN and Al_2_O_3_) into epoxy resin. The mixing is performed using a mechanical stirrer at ambient temperature for 25 min for each material to ensure even distribution. Subsequently, the appropriate hardener is added in a stoichiometric ratio, followed by an additional stirring period of at least 5–10 min. This procedure is repeated for each type of nanofiller used. The homogeneous nanocomposite mixture is poured into silicon moulds prepared with a silicone spray coating. The filled moulds are left to cure for a day at room temperature. After the curing process, the designed composites are carefully de-moulded. This methodology is consistently applied to fabricate all filler-incorporated palm/epoxy nanocomposites examined in this study. The fabricated nanocomposite samples are shown in Fig. 2c.

### Surface morphology analysis

Morphology of the treated and untreated PFFs is also explored at the HR-SEM level, specifically a Zeiss Evo10 SEM operating at a voltage of 10 kV. Before examination, the surface is covered with a sputtered gold coating.

### Thermal conductivity test

**Fig. 3:**
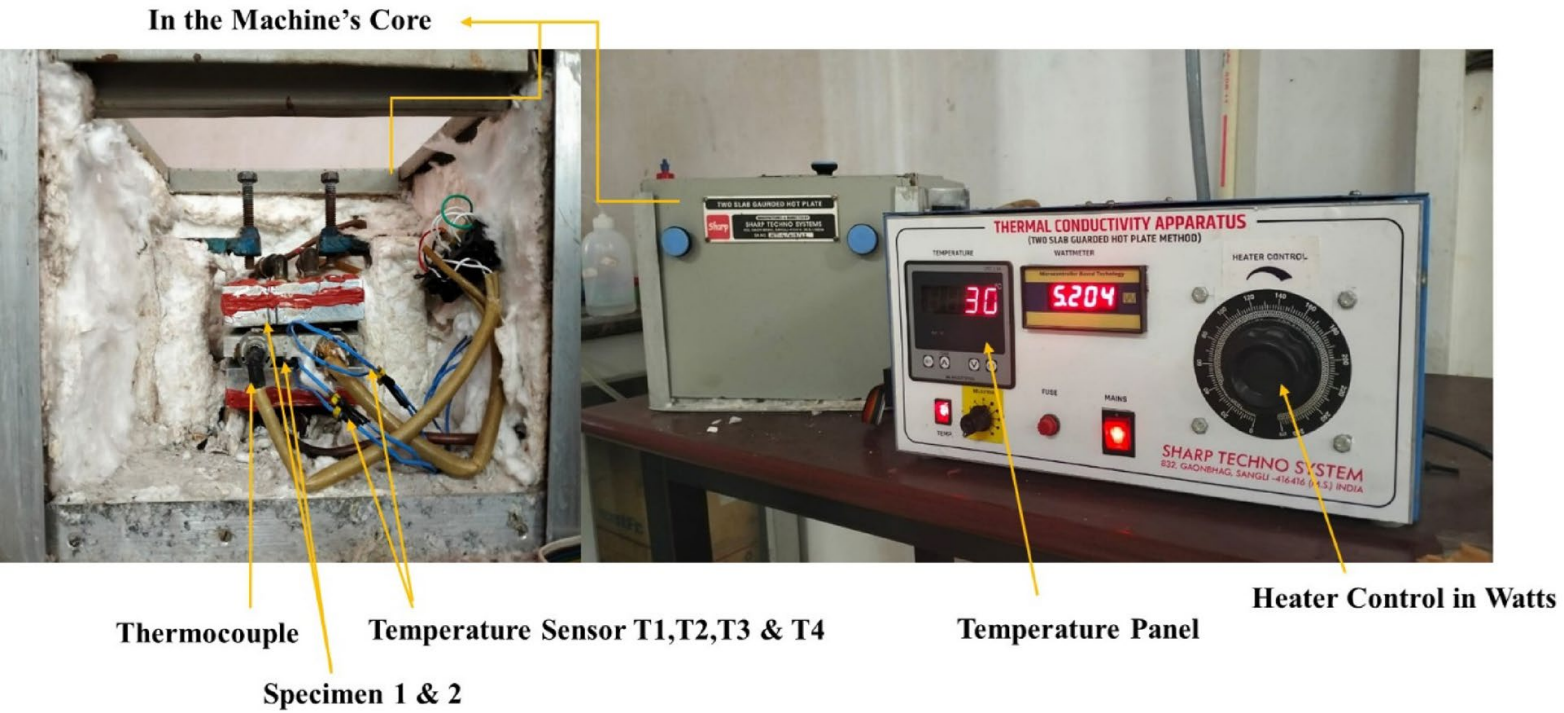
Two slab guarded hot plate equipment.

Two-Slab Guarded Hot Plate Equipment is used to compute the thermal conductivity of the epoxy-PF nanocomposite as shown in Fig. 3. To conduct the test, specimens of size 50*50 mm^2^ area and 10 mm thickness with both sides having flat surfaces are used. The Eq. (2) is used to calculate the material’s thermal conduction performance.

2 $$\varnothing =\frac{\updelta .\text{A }({T}_{h}-{T}_{c})}{\Delta z}$$

where, $$\emptyset = The \;heat\;flow\; rate\; in\; Watts$$

$$\updelta =Thermal Conductivity of the material in W/m.k$$

$$\text{A}=\text{Area of heat flow}$$

$${T}_{h}=Hot side temperature$$

$${T}_{c}=Cold side temperature$$

$$\Delta z=Thickness of used material$$

## Methodology

The response surface method / box-behnken model analysis is adopted to evaluate the impact of fiber mesh size (75–225 microns), fiber weight percentage (1–3 wt.%) and the type of nanofiller (1 wt.% of Nano MWCNT, h-BN, or Al_2_O_3_) on the thermal conductivity of palm fiber/nanofiller/epoxy composite materials. The independent factors are synthesised from literature review and initial experimental studies^40–43^. In this study, the response variable is the thermal conductivity. The three independent factors, namely fiber weight percentage, fiber mesh size and nanofillers, are labelled as A, B and C, respectively, in Table 2.

**Table 2:** Experimental range and levels of the independent variables.

Variable	Parameter	Unit	Level		
	Factor		−1	0	1
A	PF Weight Content	%	1	3	5
B	PF Mesh Size	µm	75	150	225
C	Nanofiller	%	MWCNT(1)	h-BN(2)	Al_2_O_3_ (3)

Table 3 gives the summary of total experimental runs on this research. The design is constructed using Design—Expert 13 (https://www.statease.com/software/design-expert/), a statistical software, by using a BBD. In all, 15 different combinations are generated and conducted in a randomized order of experimental trials.

**Table 3:** Sample formulations based on the Box-Behnken experimental design.

Run no	Sample name	The variable’s actual level		
		Fiber loading (%)	Fibre mesh size (µm)	Nanofiller (%)
1	P1M75N2	1	75	2
2	P5M75N2	5	75	2
3	P1M225N2	1	225	2
4	P5M225N2	5	225	2
5	P1M150N1	1	150	1
6	P5M150N1	5	150	1
7	P1M150N3	1	150	3
8	P5M150N3	5	150	3
9	P3M75N1	3	75	1
10	P3M225N1	3	225	1
11	P3M75N3	3	75	3
12	P3M225N3	3	225	3
13	P3M150N2	3	150	2
14	P3M150N2	3	150	2
15	P3M150N2	3	150	2

### Statistical analysis

Using the Mean Comparisons Test, ANOVA is employed to evaluate the differences between selected parameters based on the thermal conductivity data collected, with a significance level of *p* < 0.05 for all tests. The thermal conductivity responses are mathematically correlated through statistical analyses, utilizing multiple regression and ANOVA. A nonlinear regression model is developed using the data from the Box-Behnken Design (BBD) experiments. A significance test assesses the impact of various parameters and their interaction terms on the thermal conductivity of the epoxy/PF nanocomposite. RSM is applied to explore the relationships between multiple experimental variables and one or more response variables. The second-order polynomial response surface models are developed as shown in Eq. (3).

3 $$\left(Y\right)= {\delta }_{0 }+ \sum_{i=1}^{k}{\delta }_{i }{\varepsilon }_{i} + \sum_{i=1}^{k}{\delta }_{ii} {\varepsilon }_{j}^{2} + \sum_{i\le 1}^{k}\sum_{j}^{k}{\delta }_{ij}{\varepsilon }_{i }{\varepsilon }_{j }+ e$$

where, “y" is the predicted response, ${\delta }_{0}$ and $e$ are constant terms and error, ${\delta }_{i}$ is the linear coefficients, ${\delta }_{ii}$ is the quadratic coefficients, ${\delta }_{ij}$ is the interaction coefficients and ${\varepsilon }_{i }{\varepsilon }_{j}$ are the coded values of the independent variables, respectively.

## Results and discussions

### Alkaline treatment modification on fiber surface

**Fig. 4:**
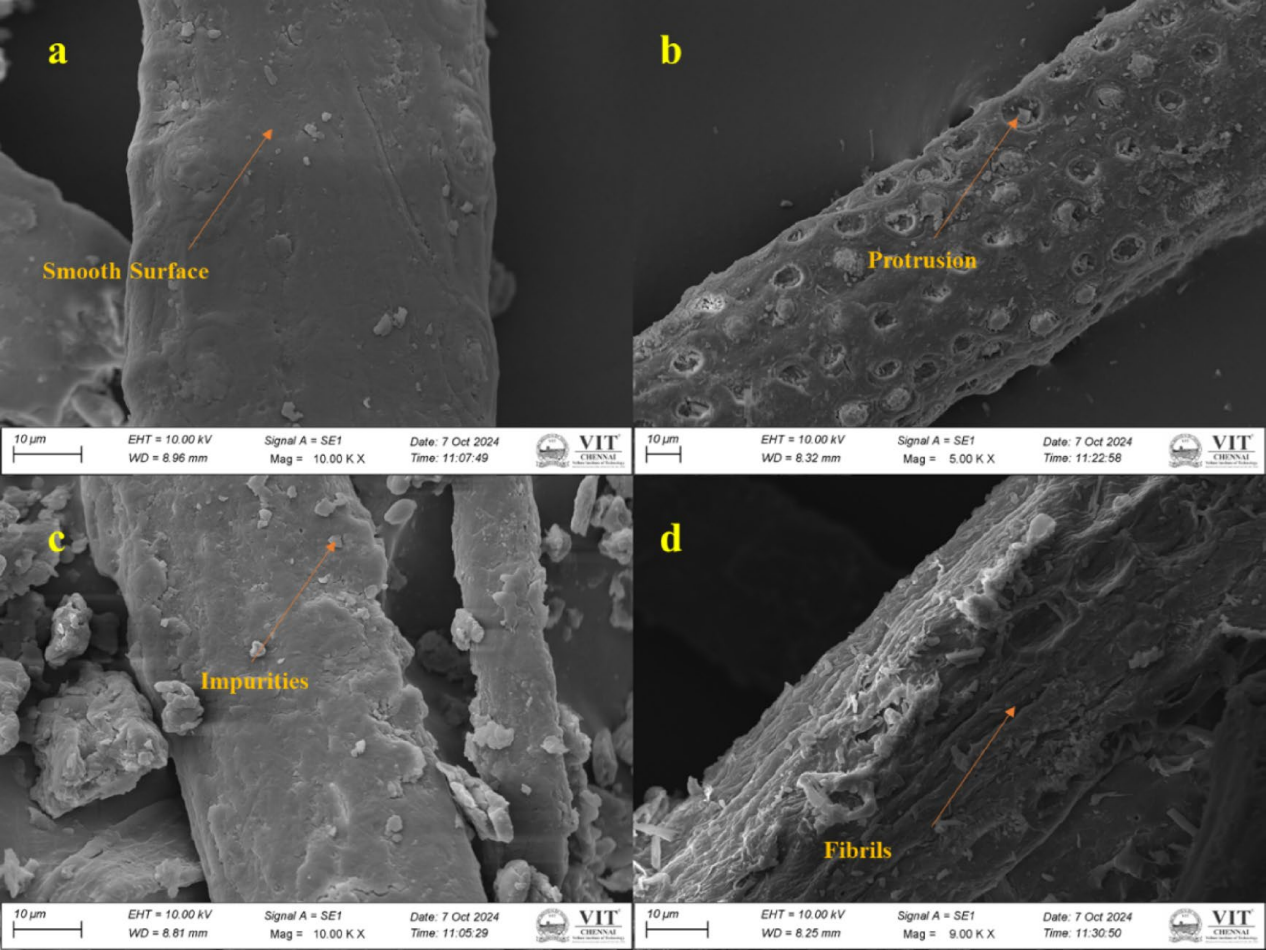
SEM analysis of fiber (**a**, **c**) untreated (**b**, **d**) treated.

The analysis of palm fruit fibers using SEM shows that there are clear distinctions between the untreated and NaOH-treated fiber specimens. Untreated fibers have a very smooth surface, marred by impurities such as wax, lignin and hemicellulose, preventing an efficient interface bond with matrix materials as shown in Fig. 4a, c. By contrast, the NaOH-treated fibers are endowed with significant surface alterations including protrusions and fibrillation as shown in Fig. 4b, d. Alkali treatment removes surface impurities and non-cellulosic constituents to reveal cellulose fibrils and increases surface fractality^44^. Increased surface area and mechanical interlocking capability of fibers is thus achieved, which will eventually lead to better bonding with matrices in composite applications. Thus, it denotes that NaOH treatment is worthy to reinforce and optimize palm fruit fibers for applications.

### ANOVA and regression model

The experimental test results, based on the formulation of the samples, are used to develop a mathematical model that describes thermal conductivity as a function of the independent variables. Table 4 is the ANOVA table for thermal conductivity with details of the sum of squares and mean square for each parameter.

**Table 4:** ANOVA for thermal conductivity.

Source	Sum of Squares	Df	Mean Square	F-value	*p*-value	
Model	0.1041	9	0.0116	13.61	0.0051	Significant
A-Fiber Weight Content	0.0265	1	0.0265	31.12	0.0026	
B-Fiber Mesh Size	0.0098	1	0.0098	11.53	0.0193	
C-Nanofiller	0.0200	1	0.0200	23.53	0.0047	
AB	0.0100	1	0.0100	11.76	0.0186	
AC	0.0049	1	0.0049	5.76	0.0615	
BC	0.0009	1	0.0009	1.06	0.3507	
A^2^	0.0028	1	0.0028	3.29	0.1297	
B^2^	0.0039	1	0.0039	4.59	0.0851	
C^2^	0.0283	1	0.0283	33.26	0.0022	
Residual	0.0043	5	0.0009			
Lack of Fit	0.0017	3	0.0006	0.4231	0.7581	Not significant
Pure Error	0.0026	2	0.0013			
Cor total	0.1084	14				
			R^2^ = 96.08%	Adj.R^2^ = 89.02%		Pre.R^2^ = 70.24%

The *p*-value and F-value are given by the ratio of mean square effect to mean square error. The "Lack of Fit" for the thermal conductivity is insignificant since its *p*-value is 0.7581, thus showing that the fitted model is good enough. In terms of the variables, the predictive model is represented as Eq. (4).

4 $$\begin{aligned} & Thermal \;Conductivity = 1.27938 + - 0.0025 * A + - 0.000133333 * B + 0.3225 * C \\ & \quad + 0.000333333 * AB + - 0.0175 * AC + 0.0002 * BC + - 0.006875 * A \wedge 2 + - 5.77778e - 06 * B \wedge 2 + - 0.0875 * C \wedge 2 \\ \end{aligned}$$

According to the obtained ANOVA outcomes, it can be concluded with 95% confidence that the regression model used in this study is suitable for the experimentation data. The main effect *p*-values and the interaction *p*-values in the predictive model are then employed to judge the significance. Any of these variables with a *p*-value less than 0.05 are considered significant for the results, implying they influence the response significantly and, therefore, the associated null hypothesis stands rejected.

Further exploration of the regression model featured computations of the R^2^, adjusted R^2^ and predicted R^2^ values. R^2^ indicates the proportion of variation in the response variable due to the model. The higher the R^2^, the better the fit of the model; it also ensures the reliability of the coefficients derived. Adjusted R^2^ commonly avoids errors for over-fitted models with an additional predictor included by adjusting against the number of terms in models. Predicted R^2^, however, evaluates the model’s ability to predict unknown new responses based on external data that is not used for fitting the model. The comparison would be also considered better between the two models.

The model’s goodness-of-fit is determined via R^2^ and adjusted R^2^ coefficients. A maximum difference of 0.20 remains alluded to a credible explanation; a large contrast could represent complications with the data or model. For this study, the predetermined values using the prediction R^2^ and adjusted R^2^ for thermal conductivity are 0.7024 and 0.8902, respectively; they depict reasonable agreement and harmony between the predicted and observed responses. The coefficient of determination R^2^, which is equal to 0.9608, reflects the model’s ability to explain 96.08% of the variability of the experimental data. Limited, these measures presently show a good correlation between the measured and predicted values, thus confirming the robustness of the model.

### Analysis of residual plots of nanocomposite

**Fig. 5:**
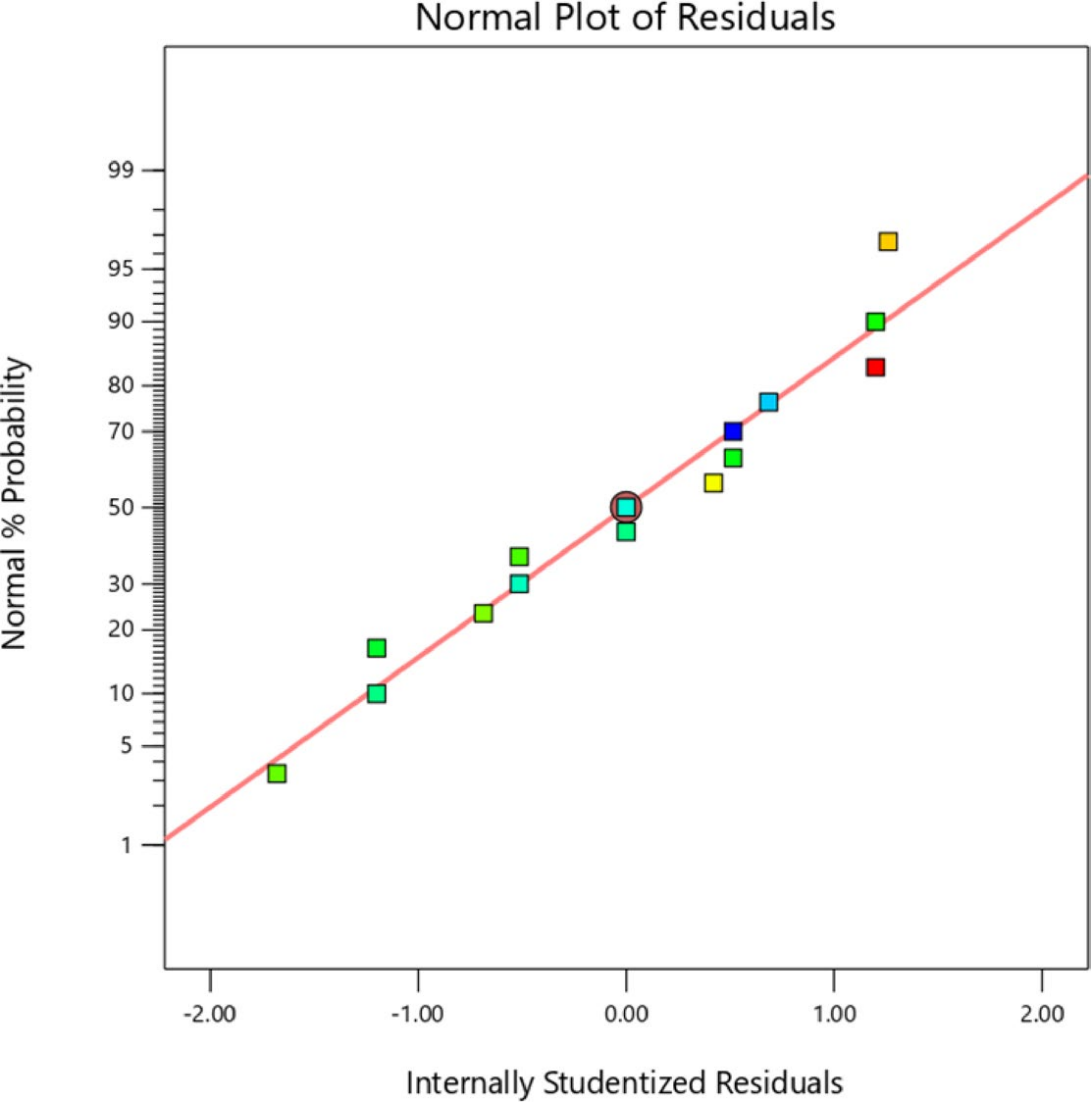
Residual thermal conductivity formulated in terms of the normal probability plot.

Furthermore, Fig. 5 depicts the normal possibility plot of the thermal conductivity residuals, which indicates a straight-line trend. This linear distribution suggests that the residuals are regularly distributed, which implies that the model is appropriate. The alignment of residual points along the straight line in these figures demonstrates the model’s adequacy. Further, verification of the model’s correctness in reflecting the experimental data is performed by analysing the residuals’ behaviour using regression.

**Fig. 6:**
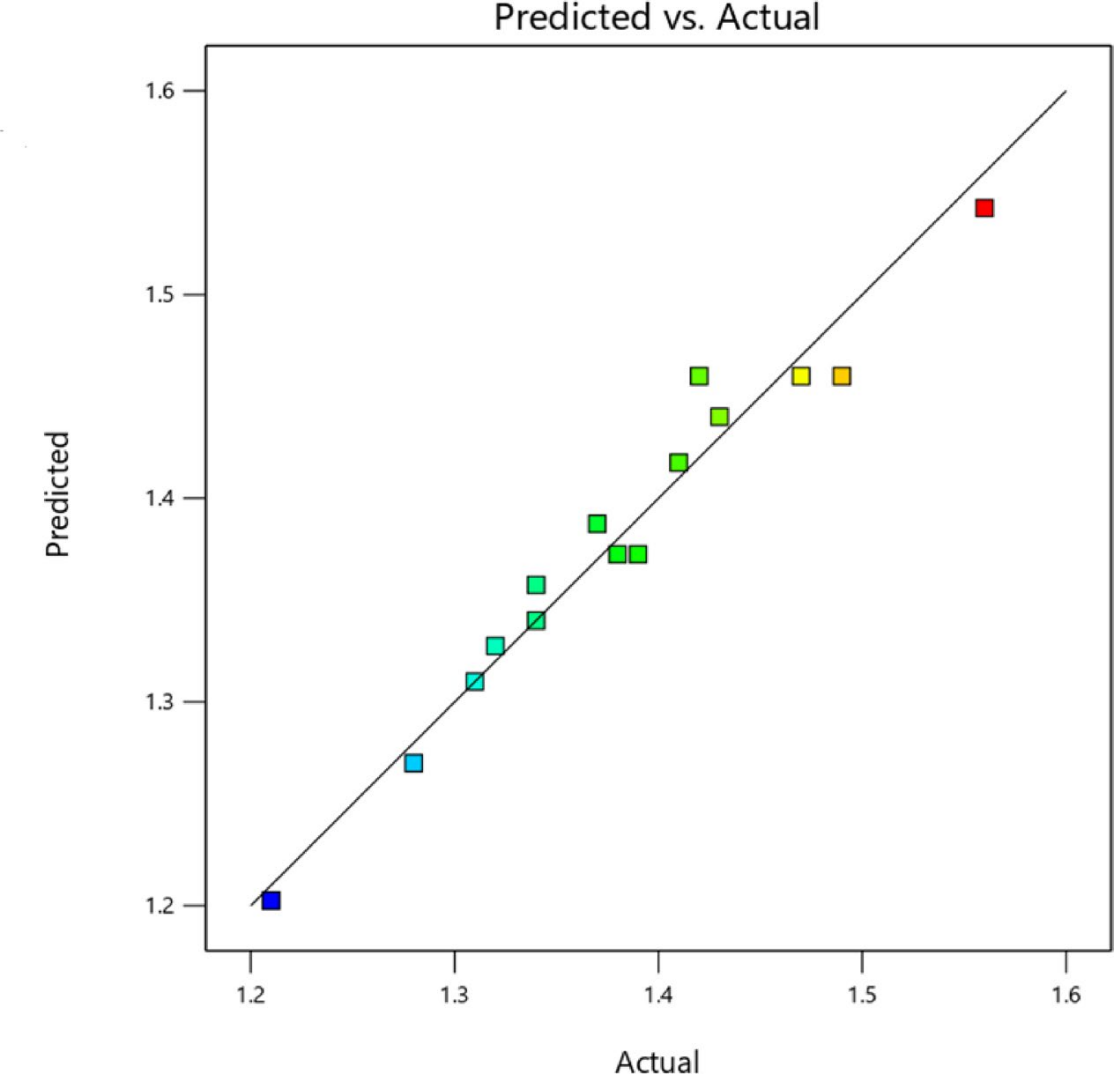
Thermal conductivity is described by graphs of predicted vs actual values.

An adequate model can be evaluated by examining the distribution of statistics points about the mean of the response variable. A uniform distribution of data points around the mean indicates model adequacy as shown in Fig. 6. The relationship between the predicted response values from the quadratic model equation and the actual experimental response is analysed using a predicted versus actual plot. This plot demonstrates a strong correlation, with an R^2^ value of 0.9608 for thermal conductivity, indicating that the model effectively describes the experimental data. Additionally, Table 5 compares the measured values with their corresponding predicted values, showing a maximum error of 2.8% for thermal conductivity.

**Table 5:** Thermal conductivity comparison of measured and predicted values.

Sample name	Response of thermal conductivity		Error %
	Actual	Predicted	
P1M75N2	1.56	1.543	−1.12
P5M75N2	1.32	1.328	0.57
P1M225N2	1.38	1.373	−0.54
P5M225N2	1.34	1.358	1.31
P1M150N1	1.41	1.418	0.53
P5M150N1	1.39	1.373	−1.26
P1M150N3	1.37	1.388	1.28
P5M150N3	1.21	1.203	−0.62
P3M75N1	1.43	1.440	0.70
P3M225N1	1.32	1.340	1.52
P3M75N3	1.29	1.310	1.55
P3M225N3	1.28	1.270	−0.78
P3M150N2	1.47	1.460	−0.68
P3M150N2	1.42	1.460	2.82
P3M150N2	1.49	1.460	−2.01

**Fig. 7:**
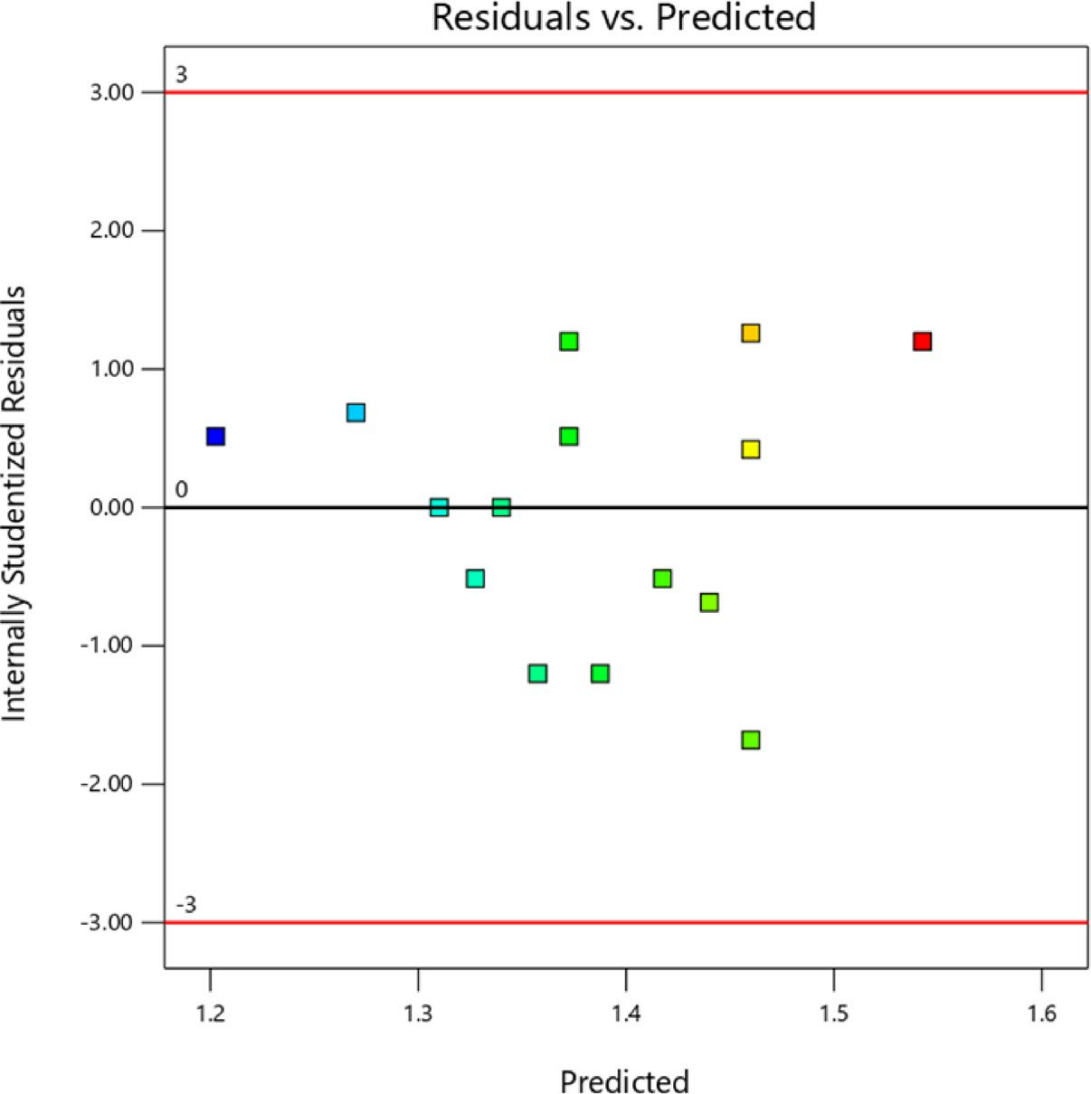
Residual plot for Thermal Conductivity vs Predicted Values.

Figure 7 depicts the residuals versus expected plot for the thermal conductivity test, which shows that the model is adequate. The residuals are randomly spreaded around the zero line, with no discernible design, implying errors are evenly distributed and that the model’s assumptions, such as constant variance and unbiased predictions, are valid. In addition, most of the residuals are within acceptable limits, with few outliers or areas of concern. The close alignment of actual and anticipated values adds to the model’s reliability, indicating that it delivers an accurate and appropriate representation of thermal conductivity data.

### Surface plots for thermal conductivity

This section uses 3D and 2D surface plots to demonstrate the effect of design factor parameters on thermal characteristics. These plots are useful for assessing the combined impact of the key variables on the responses based on investigation.

Fillers and reinforcements considerably impact polymers’ thermal characteristics. Filler particle dispersion, fibre orientation, relative stiffness of fibre and matrix, aspect ratio and thermal expansion misalliance impact the thermal behaviour of composite^41^. Table 5 shows the measured thermal conductivity values of epoxy/palm composites and their associated errors, for three different fibre loading levels.

### Effect of fiber wt. % with fiber mesh size

**Fig. 8:**
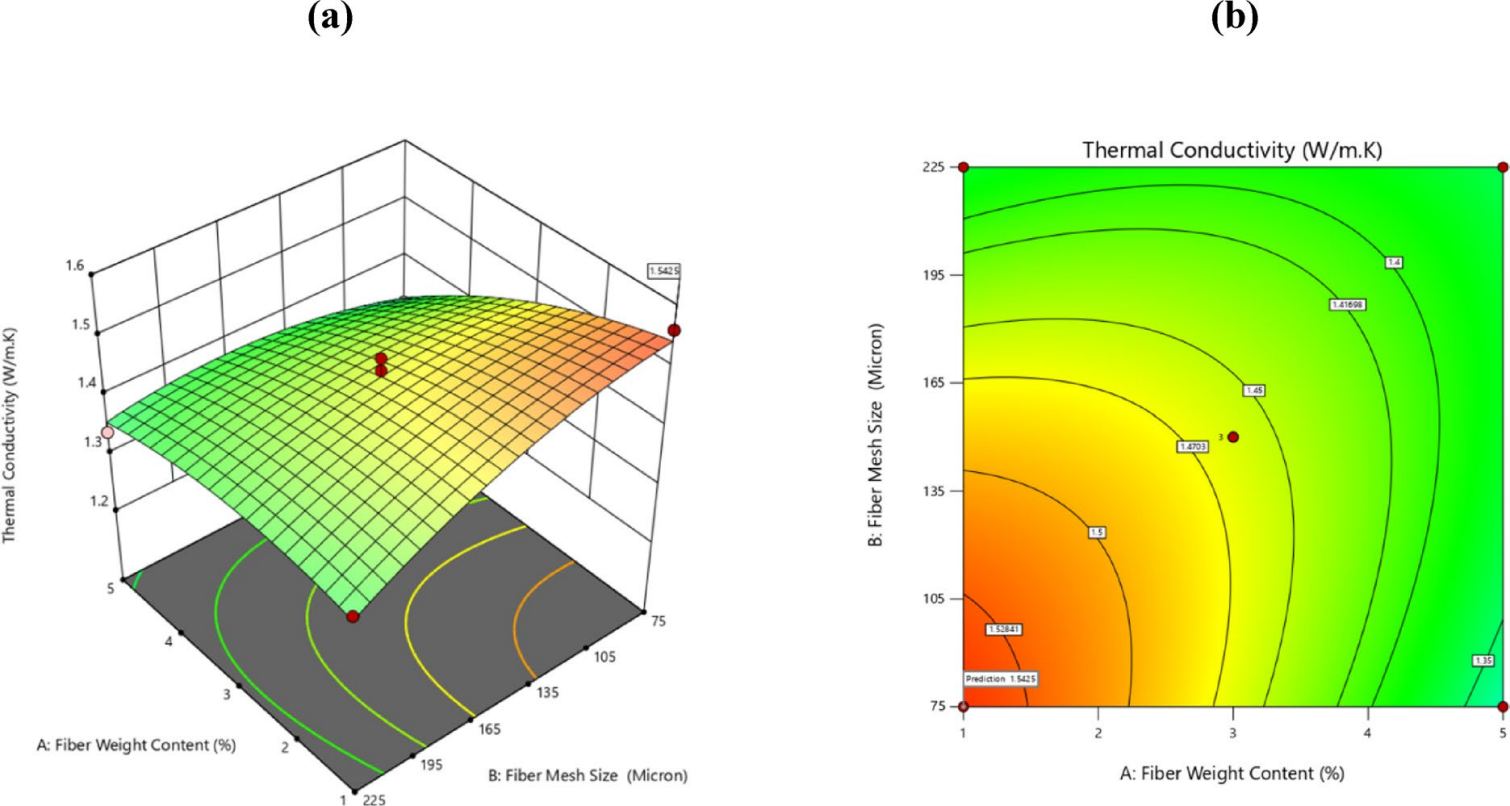
Plotting of the effects of fiber weight content and fiber mesh size in 3D and 2D.

Figure 8a, b presents 3D and 2D surface response plots illustrating the influence of fiber loading and fiber mesh size on thermal conductivity. It is illustrated in Fig. 4a, b that the increase in palm fiber loading leads to reduction in thermal conductivity. This occurs due to the hollow fibrous cellular structure acting as a thermal insulator within the composite, thereby reducing thermal movement. Moreover, using palm fiber in the epoxy matrix reduces thermal conduction. Besides, –OH groups in cellulose form internal and external hydrogen bonds due to their structure therefore these groups do not conduct heat as efficiently as the epoxy matrix^41,45^.

The smaller meshes when employing organic fibers can increase their surface area and promote a better fiber dispersion rate in the polymer, leading to improved fiber-matrix interaction and bonding which will modify the thermal properties of the final composite. Factors like composition, structure and degree of orientation of fibers affect the thermal conductivity of composite materials. Smaller mesh sizes allow greater volume fraction of fibers to be packed which allows the desired properties of the fibers to be realized in thermal performance more than (with) coarse fibers^46^. This improvement can be owed to two reasons, first, the contact area, as more fiber surface area allows for better heat conduction across interfaces and second the air gaps, because finer fibers reduce gaps filled with air which is a poor conductor and therefore improves heat transfer in the composite^47^.

### Effect of fiber loading with nanofiller

**Fig. 9:**
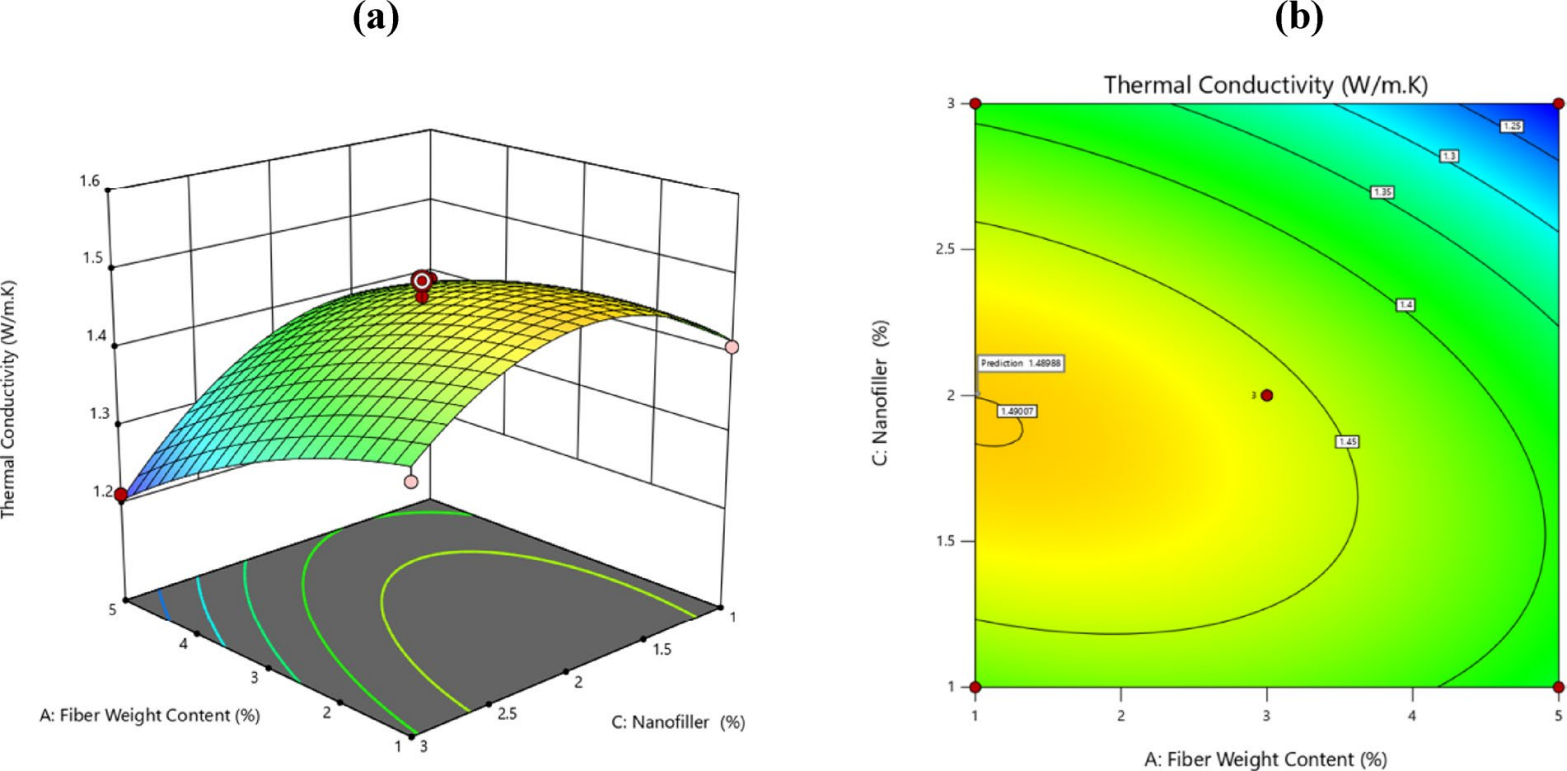
Plotting of the effects of fiber loading and nanofiller in 3D and 2D.

The dual influence stemming from the loading of natural fibers together with the addition of BN nanofillers leads to clear changes in thermal conductivity. It is proved in studies that the combination of natural fibers and BN has a greater impact on thermal properties than each component alone as shown as the Fig. 9a, b.^20^. Thus, the use of BN in addition to natural fibers results in the development of more efficient thermal networks within the composite structure, owing to improved interactions between the fibers and nanoparticles. The presence of Nano-BN in composite materials significantly enhances their thermal conductivity, which is advantageous for applications where effective heat dissipation is essential.

According to the latest studies, the loading levels of natural fibers changes the thermal properties of the composites produced. Mechanical properties generally tend to improve with increased fiber loading, while thermal stability may decrease^48^. On the other hand, the best possible loading of BN nanofillers increases thermal conductivity significantly, whilst mechanically reinforcing the material. It is advisable that both fiber content and BN loading are optimized for thermal and mechanical behaviour, as well as issues such as BN material agglomeration that can affect performance^20,49^.

### Effect of fiber mesh size with Nanofiller

**Fig. 10:**
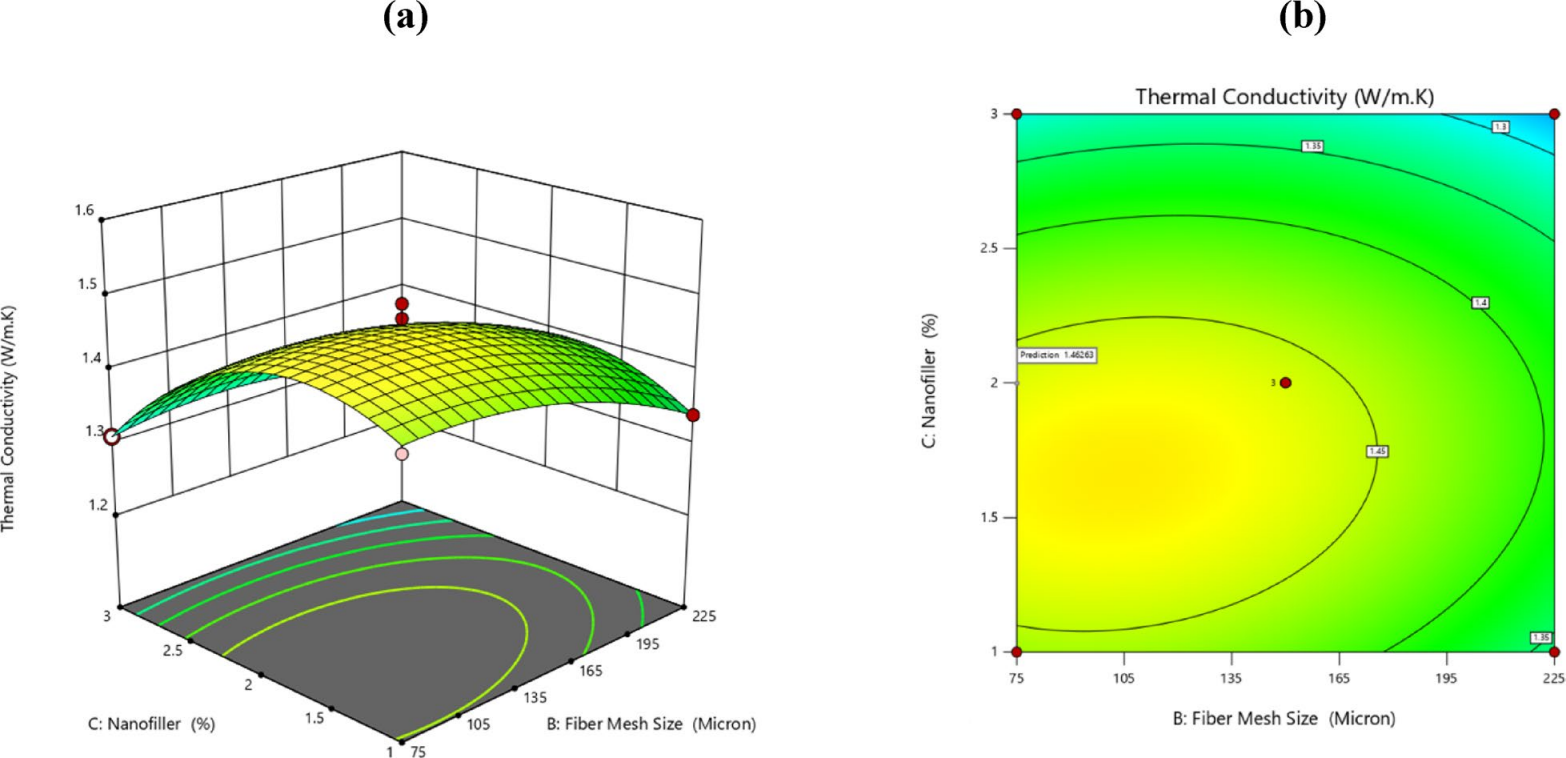
Plotting of the effects of fiber mesh size and nanofiller in 3D and 2D.

Graph (Fig. 10) indicates that the maximum thermal conductivity value occurs when the fiber mesh size is low (more fine fibers) and the h-BN nanofiller content. The reason for this is, better composite density and interfacial bonding can be achieved due to the presence of finer fibers whereas due to their high thermal conductivity, BN nanofillers add pathways for heat conduction. The interplay of these factors results in the overall improvement where more thermal conducting pathways are present and the network is less porous as shown in the highest point on the surface plot.

### Optimal condition

**Fig. 11:**
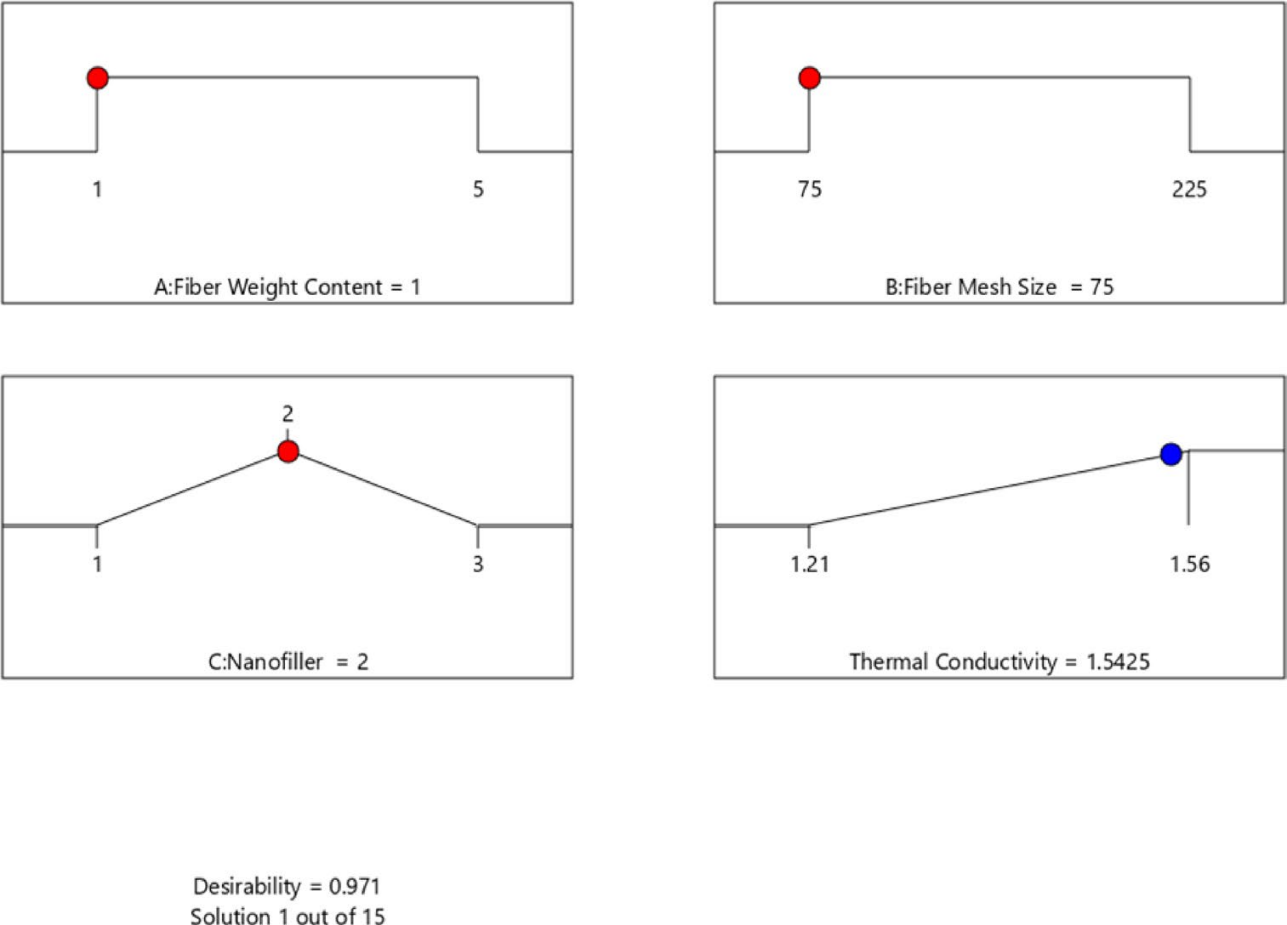
Numerical optimization graph of ramp for variables A, B, and C.

Developing fifteen solutions in the software makes it possible to select one as the optimal solution at maximum thermal stability. Red indication points to the optimal conditions (fiber content, fiber mesh size and nanofiller) to obtain the maximum thermal conductivity factor which is given by the blue dot and the desirability of the solutions is close to 1 (0.971), as presented in Fig. 11. The desired values of all independent and dependent parameters due to optimization using a desirability function are presented in Table 6.

**Table 6:** Validation of optimal solution in the desirability space.

	Fiber content (%)	Fiber mesh size (µm)	Nanofiller	Thermal conductivity (W/m.K)	Desirability
RSM prediction	1	75	h-BN (1%)	1.54	0.971
Experimental				1.51	-
% Error	-	–	–	1.99	-

The graph (Fig. 11) depicts the optimum combination of parameters with a significantly high desirability of 0.971 and thermal conductivity of 1.54 W/m·K. The optimal parameters are as follows: fiber weight content (A) kept at its minimum threshold of 1, fiber mesh size (B) not greater than 75 microns and the percentage of nanofiller content (C) equal to 1% of BN. This is to take advantage of a low-density structural fiber-weight distribution and good interfacial bonds with no fillers consisting of coarser fibers, as well as evenly distributed, non-agglomerated BN nanofillers, to enhance thermal conductivity effectively.

**Fig. 12:**
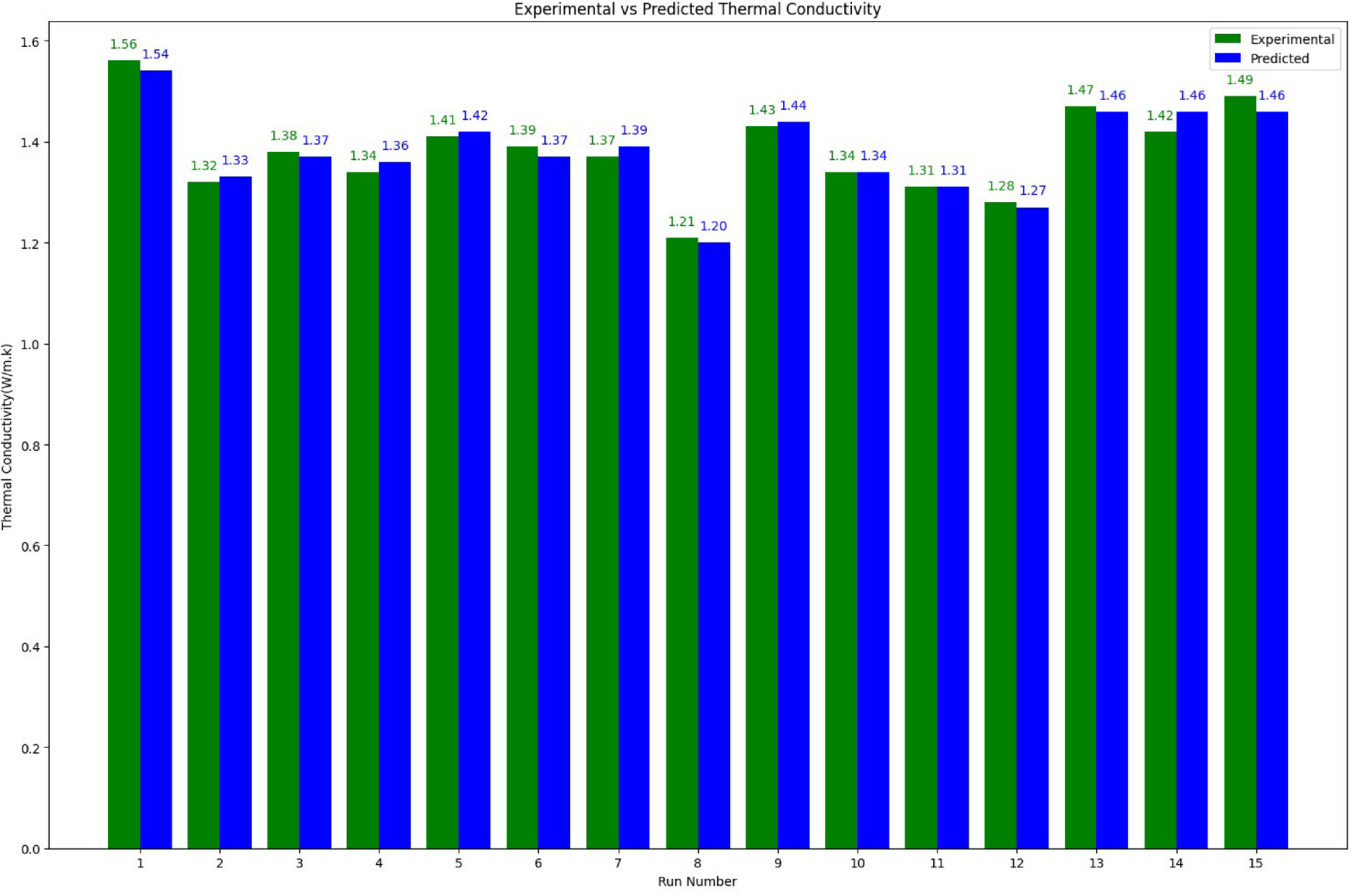
Thermal conductivity of experimental and predicted.

Besides, the thermal conductivity experiment is repeated under optimum conditions to compare the accuracy of the predicted responses by RSM model with the experimental results. Such optimized yields define responses as shown in Table 6, namely, fiber content, fiber mesh size and nanofiller. Errors between experimental results and Response Surface Methodology predictions can be noted as 1.32% for thermal conductivity. Thus, both results have a good agreement, which implies accurate predictions by RSM model. Figure 12 shows the thermal conductivity of experimental and regression responses. Most of the predictions tend to correlate with the experimental results, attesting to the model being able to predict accurately.

### Comparative study

Table 7 compares thermal conductivity values of various fiber-reinforced composites. This study’s palm fruit fiber/epoxy composite with 1 wt.% h-BN attained a thermal conductivity of 1.54 W/m·K, exceeding systems such as Kevlar/Al_2_O_3_ (0.6376 W/m·K and rice husk/MWCNT (0.2577 W/m·K).It outperforms PVA/CNC (0.74 W/m·K) and natural rubber reinforced with BN/lignosulfonate (1.17 W/m·K), indicating its potential for thermally demanding applications.

**Table 7:** Comparative analysis of thermal conductivity.

S. no	Matrix	Reinforcement	Filler	Thermal conductivity (W/mK)
1	Epoxy	Palm Fruit Fiber	hBN (1 wt.%)	1.54 (Present Study)
2	Epoxy	Kevlar	Al_2_O_3_ (3 wt.%)	0.6376^50^
3	Epoxy	Rice Husk	MWCNT (1 wt.%)	0.2577^51^
4	Natural rubber	–	MWNT (1 wt.%)	0.4^52^
5	Polypropylene	–	CNF (50 wt.%)	3.46^53^
6	PVA	–	CNC	0.74^54^
7	Epoxy	Kenaf	hBN (43.6 wt.%)	6.418^20^
8	Epoxy	–	SiC@CFA	0.404^18^
9	Natural rubber	–	BN/lignosulfonate	1.17^22^
10	Polycarbonate	-	GNP (20 wt.%)	1.8 & 7.3^55^

## Conclusion

The research on palm fiber content, mesh size of the palm fiber and type of nanofiller is conducted about its effect on thermal conductivity in the case of epoxy-based composites. Alkali treatment effects on surface morphology are studied in Palm fiber. SEM exhibits that significant chemical modifications are recorded on the surfaces and shows that alkali treatment leads to the removal of waxes and other surface impurities. Optimal systematic investigations on process parameters is done using Box-Behnken Design (BBD). The factors investigated include the palm fiber percentage in the composites (1–5%), the mesh size (75–225 µm) and the nanofiller type (MWCNT, h-BN and Al_2_O_3_). It is found that the thermal conductivity is greatly affected by the above parameters, with the type of nanofiller and palm fiber mesh size having a pronounced effect. The optimal conditions for maximum thermal conductivity are 1wt. % palm fiber content, 75 µm mesh size and 1% hexagonal boron nitride as the nanofiller. For these conditions, thermal conductivity is found to be 1.54 W/m·K. The experimental data nicely matches with the predicted values showing the robustness of the regression model developed. This study confirms the significant performance of the use of BBD in optimization of composite properties and the development of high-performance epoxy composites. It would speculate toward very useful advanced materials with better thermal conductivity for various industrial applications.

## Data Availability

The datasets used and/or analysed during the current study available from the corresponding author on reasonable request.
